# Exercise Prescription for Postprandial Glycemic Management

**DOI:** 10.3390/nu16081170

**Published:** 2024-04-14

**Authors:** Alessio Bellini, Alessandro Scotto di Palumbo, Andrea Nicolò, Ilenia Bazzucchi, Massimo Sacchetti

**Affiliations:** Department of Movement, Human and Health Sciences, University of Rome “Foro Italico”, Piazza Lauro De Bosis 6, 00135 Rome, Italy; alessiobellini1@gmail.com (A.B.); a.scottodipalumbo@fondazioneuniroma4.it (A.S.d.P.); andrea.nicolo@uniroma4.it (A.N.); massimo.sacchetti@uniroma4.it (M.S.)

**Keywords:** post-meal exercise, exercise timing, exercise volume, postprandial glycemia, activity breaks, exercise type, diabetes

## Abstract

The detrimental impacts of postprandial hyperglycemia on health are a critical concern, and exercise is recognized a pivotal tool in enhancing glycemic control after a meal. However, current exercise recommendations for managing postprandial glucose levels remain fairly broad and require deeper clarification. This review examines the existing literature aiming to offer a comprehensive guide for exercise prescription to optimize postprandial glycemic management. Specifically, it considers various exercise parameters (i.e., exercise timing, type, intensity, volume, pattern) for crafting exercise prescriptions. Findings predominantly indicate that moderate-intensity exercise initiated shortly after meals may substantially improve glucose response to a meal in healthy individuals and those with type 2 diabetes. Moreover, incorporating short activity breaks throughout the exercise session may provide additional benefits for reducing glucose response.

## 1. Introduction

The adverse impacts of glucose variability and postprandial glucose spikes have undergone extensive scrutiny in recent years [1,2,3]. A significant portion of the day is spent in the postprandial state by most individuals [4,5], which amplifies the risks linked to prolonged high blood glucose levels. Notably, markedly excessive post-meal glucose spikes have been linked to increased oxidative stress, inflammatory markers, and endothelial dysfunction in both healthy individuals and those with diabetes. These conditions have been associated with an elevated susceptibility to developing cardiometabolic disorders and complications related to diabetes [1,3,6,7,8,9]. Nutrition and exercise are crucial as primary interventions to mitigate excessive glycemic responses in both healthy people and people with diabetes [10,11,12,13,14]. Nevertheless, despite the well documented benefits of exercise, sedentary behaviors are alarmingly on the rise across populations, contributing to an increased incidence of cardiometabolic disorders [15,16,17,18,19]. Therefore, incorporating regular physical activity and post-meal exercise is crucial for reducing the adverse effects of sedentary behaviors and managing blood glucose fluctuations.

Numerous studies have previously highlighted the advantages of exercise in regulating post-prandial blood glucose levels [10,11,12,13,14]. The American College of Sports Medicine (ACSM) has recently reiterated the significance of postprandial exercise for individuals with type 2 diabetes (T2D), recommending at least 45 min of any exercise type, at any intensity, after meals to improve postprandial glucose response [20]. This represents a significant step toward identifying specific exercise prescriptions for optimal post-meal glucose responses. However, fine-tuning modulation of exercise for maximum benefit remains a complex challenge.

Hence, this review aims to refine exercise prescriptions by examining the influence of various exercise parameters on post-meal glucose responses in both healthy individuals and those with T2D. By exploring the effects of exercise timing, volume (including duration and intensity), type, and pattern on post-meal glucose response, we could provide practical indications for tailoring exercise to enhance glucose management after meals for people with T2D and without.

## 2. Is There an Optimal Time for Exercise Relative to Meals? The Effects of Exercise Timing on Postprandial Glucose Response

The relationship between the timing of physical exercise and meals has garnered increasing attention for its potential to improve blood glucose levels after eating [10,12,13,14,21]. Given the crucial role of timing in exercise prescriptions, it has been suggested to introduce a third “T” into the FITT principles (frequency, intensity, time, and type) to emphasize this [22]. Furthermore, the latest physical activity guidelines for individuals with T2D recommend post-meal exercise for optimizing control over postprandial glucose excursions [20], echoing evidence that post-meal exercise significantly influences glucose management in healthy and diabetic individuals [10,11,12,14,21,23,24]. Although pre-meal exercise has been shown to attenuate postprandial lipid responses, particularly when performed several hours before eating [11,12], its impact on acute blood glucose management is less significant, especially if performed 12–16 h before eating [25,26]. Conversely, moderate-intensity aerobic or resistance exercises conducted 20–45 min prior to a meal have been shown to markedly lower post-meal glucose levels [27,28]. Similarly, Francois et al. (2014) reported improvements in the glucose response to each daily meal among individuals with impaired glucose tolerance and/or T2D who engaged in intense “exercise snacks” 30 min before meals [29]. These findings suggest benefits in timing exercise closely with meals. However, post-meal exercise seems to elicit a greater management of blood glucose compared with pre-meal exercise in healthy individuals and those with T2D [23,30,31,32,33,34], offering a significant reduction in the post-meal glucose excursion and the overall glycemic response [24,35,36]. The different effects between pre-prandial and post-prandial exercise may be influenced by several factors, such as the individual’s nutritional status, exercise intensity, and volume. These elements play critical roles in determining the efficacy of exercise in modulating glucose responses at a specific time.

Exercise prescriptions, therefore, consider not only the sequence of meals and exercise but also the time interval between them in relation to the expected blood glucose response. In healthy individuals, the glucose peak occurs 30 to 60 min after eating [9]. Research findings vary, with some studies reporting significant attenuation of the glucose peak when exercising 30 or 45 min after the start of the meal [37], while others have demonstrated that commencing exercise shortly after a meal more effectively blunts post-meal glucose excursion [23,28,34]. In particular, beginning exercise around 10–15 min after eating may attenuate the post-meal glucose peak more effectively compared to starting around 30 min later [23,38]. This is particularly relevant to prevent cardiometabolic disorders, as glucose levels in healthy individuals typically remains below 140 mg/dl, a threshold often surpassed in people with T2D [12,21,39].

Conversely, in people with diabetes, peak glucose levels reach considerably higher values, often occurring between 60 and 120 min after a meal [12,21,39]. This necessitates different considerations for optimal exercise timing prescriptions. Consistent with findings in healthy individuals, a broad consensus among studies suggests substantial benefits of exercising soon after a meal in people with T2D [12]. These studies demonstrate that exercising before the peak glucose levels occur yields greater benefits than waiting for longer periods post-meal [24,40]. For instance, 15 to 30 min of aerobic or resistance exercises initiated 30 min after a meal significantly reduced post-meal glucose peaks [24]. Similarly, a notable reduction in glucose excursions has been observed when exercise begins 45 min after breakfast or dinner [27,41], with some studies noting improvements even when activity commences 15 to 20 min after the start of a meal [35]. Despite these insights, further research is required to determine the optimal exercise timing for people with diabetes.

Considering all this evidence, initiating postprandial exercise before the peak glucose levels occur, approximately 15 min in healthy individuals and 30 min in people with T2D, is recommended. This approach may mitigate the post-meal glucose peak and overall glucose response.

In determining the optimal timing for exercise in relation to meal consumptions, it may be worth considering the specific metabolic effects induced by various options. Exercise enhances microvascular recruitment and blood flow in active muscles, thereby increasing glucose delivery, uptake, and disappearance from the bloodstream [42]. However, the nutritional state at the time of exercising may assume an important role in the dynamics of glucose appearance and disappearance.

Pre-meal exercise, especially in a fasted state, stimulates insulin sensitivity and fat oxidation, primarily sourcing glucose from hepatic sources, and in particular from glycogenolysis [42]. This mechanism aids in maintaining blood glucose stability, preventing hypoglycemia and countering the potential rise in glucose uptake induced by exercise via insulin-independent mechanisms [42]. Additionally, exercising in a fasted state may help in lowering fasting hyperglycemia and might be preferable to postprandial exercise, as it is characterized by lower gastrointestinal discomfort and may be more feasible for adopting higher intensities.

In the post-prandial state, glucose mainly derives from exogenous sources while hepatic glucose production is markedly reduced by food ingestion [42]. Glucose uptake during postprandial exercise is supported by both contraction and insulin-independent mechanisms, while the insulin response from meal ingestion is reduced [42]. With regards to the time window for postprandial exercise, it has been suggested that exercising too early after the meal may initially lower glucose levels, but could lead to a rebound increase later, diminishing the beneficial effects [12]. Therefore, it has been proposed that exercising in the so-called mid-postprandial period may represent the optimal choice for improving both the immediate post-meal glucose peak and overall daily glucose management in individuals with T2D or insulin resistance [43].

Incretin hormone (i.e., GLP-1 and GIP) secretion may be stimulated by exercise and play a role in slowing gastric emptying, thus facilitating the lowering of post-meal blood glucose concentrations [44].

One aspect to consider is that the glycemic response to the same meal can vary significantly between individuals [45], influenced by factors such as pre-exercise glucose levels, glucose tolerance, and glucose loads [46]. This variability suggests the importance of personalizing the starting time of exercise based on individual responses. In this regard, it has been suggested that initiating exercise 20 min before an individual postprandial glycemic peak can be effective [38]. This could be easily achieved by using continuous glucose monitoring (CGM), which could serve an educational purpose, helping individuals understand the impact of exercise on their glucose levels and potentially improving long-term adherence to exercise routines by providing real-time feedback on the effects of different activities and timings [47].

When discussing the optimal timing of exercise, another crucial factor to delve into is the specific time of day when physical activity occurs and its alignment with meals, which introduces significant complexity beyond the simple pre- or post-meal dichotomy. For instance, the dynamics of exercising before breakfast, in a state of fasting, vary distinctly from activity performed later in the day when the body is not in a fasted state. Research has shed light on the potential benefits of repeated exercise in a fasted state, often referred to as fasted training, which is noted for favorable adaptations in metabolic control, as highlighted in a comprehensive review by Edinburgh et al. (2022) [48].

Expanding this discussion further, it is crucial to acknowledge the influence of various factors beyond meal timing. Notably, the body’s insulin sensitivity is not static throughout the day; it diminishes as the day advances, with a significant reduction in the evening. This fluctuation could significantly impact the body’s response to exercise at different times of the day. Moreover, the post-meal glycemic response is influenced by more than just the immediately preceding meal. The “second-meal effect,” where the glycemic response to a meal is affected by earlier dietary intake and activity, underscores the interconnectedness of daily eating and exercise patterns. This effect suggests that the timing of exercise around lunch may have distinct outcomes compared to morning or evening sessions, areas that require further exploration given the concentration of research on morning and evening activities; far fewer studies have investigated the interaction between meals and exercise at lunchtime [49,50]. In addition, post-exercise nutrition, particularly carbohydrate intake, plays a critical role in enhancing insulin sensitivity, yet this does not always correlate directly with immediate postprandial glucose levels [51]. This phenomenon emphasizes the intricate interplay between the timing of meals and subsequent physical activity in shaping the metabolic responses.

Considering these multifaceted aspects provides a more comprehensive understanding of how the timing of exercise, in relation to meal schedules and the body’s circadian rhythms, can impact our metabolic responses and overall health outcomes.

In the context of diabetes, the interaction between exercise and pharmacological treatment is another relevant aspect to consider, as it may synergistically potentiate the reduction in post-meal glucose levels. However, findings on this aspect are restricted and discordant. In particular, it has been shown that diabetic individuals under metformin treatment have a significant reduction in the glucose peak after an exercise session started 30 min from the beginning of the meal [40,52]. Erikson et al. (2017) have reported that combining exercise and metformin elicited a greater reduction in the post-meal glucose response than either interventions alone [52]. Likewise, a recent study showed that engaging in 50 min of high-intensity interval exercise, initiated 90 min prior to an oral glucose tolerance test in conjunction with metformin, significantly improved insulin sensitivity compared to either intervention independently [42]. However, other research presents mixed outcomes, with some studies not observing additional benefits from combining exercise with metformin treatment, either acutely [53,54] or over the long term [55]. These findings highlight the potential for exercise to complement pharmacological treatment in diabetes management, though evidence remains varied. Further research is needed to clarify the optimal strategies for integrating physical activity with medication to maximize therapeutic benefits. Table 1 summarizes the key findings from the studies comparing different exercise timings.

## 3. Which Type of Exercise Is the Most Effective?

Identifying the most effective exercise type for managing glucose plays an important role in prescribing exercise for post-meal glucose responses. It is firmly established that both aerobic and resistance exercises, especially when combined, significantly improve long-term glucose control in individuals with diabetes [56,57]. This is supported by research demonstrating their effectiveness in reducing 24 h and postprandial blood glucose levels, underscoring their important implications for diabetes management [10,11,14,41,58]. The latest physical activity guidelines for individuals with T2D recommend engaging in any form of exercise to enhance the glucose response after a meal [20].

Over recent years, numerous studies have explored the specific impacts of aerobic and resistance exercises in isolation on post-meal glucose responses. Specifically, research has demonstrated the efficacy of various aerobic exercises in reducing the glucose response to meals in both healthy individuals and those with T2D [23,59]. For example, studies have reported that cycling at different intensities significantly diminishes post-meal glucose excursions and overall responses in both healthy and diabetic populations [23,37,60,61,62]. Likewise, walking and running have shown significant improvements in the glucose response after a meal [23,30]. Studies conducted in our laboratory have indicated that engaging in 30 min of moderate-intensity walking, jogging, or cycling similarly reduced the post-meal glucose peak and 3 h postprandial glucose concentration in healthy individuals [23]. Additionally, elliptical exercise has been effective in lowering post-meal glucose spikes in healthy individuals [23].

While walking might be the simplest exercise to undertake due to its minimal equipment requirements, many individuals may find it challenging to step away from their workplace or home. Hence, alternative exercises that require minimal space availability or specific equipment, like stair climbing and descending and step exercises, fit these requirements. Several studies have demonstrated that brief intervals of 1 to 10 min of stair climbing and descending significantly enhance the post-meal glucose response in individuals with diabetes [63,64,65,66]. Recently, our findings have supported this, showing comparable benefits between 30 min of step exercises, structured as alternating 30 s of moderate-intensity work with 60 s of rest, and a 30 min continuous walk [32]. Taken together, this evidence indicates that although walking emerges as a primary option for improving the postprandial glucose response in both healthy individuals and those with diabetes, other aerobic exercise alternatives are available to accommodate the diversity in personal preferences and requirements.

Resistance exercise, like aerobic exercise, has shown positive impacts on post-meal glucose responses in both healthy and T2D individuals. Previous studies have highlighted the beneficial impact of engaging in resistance training either before or after consuming high-carbohydrate meals, with both approaches leading to significant improvements in glucose management [67,68,69]. Both traditional and circuit resistance training have demonstrated notable reductions in postprandial blood glucose levels. For example, performing 15 to 30 min of circuit resistance training after a standardized breakfast significantly lowered blood glucose levels in individuals with T2D and healthy counterparts, respectively [23,24]. Similarly, in the same populations, three sets of 16–30 repetitions at light-to-moderate intensity resulted in a clear reduction in the post-meal glucose peak and overall glycemic response to a meal [69].

Exploration into alternative methods of training muscle conditioning, such as neuromuscular electrical stimulation (NMES), has shown promising results. NMES has been reported to decrease glycemic levels after 30–60 min of passive application applied to leg muscles in both healthy and T2D individuals [70,71]. However, the combination of NMES with voluntary muscle contractions appears necessary to harness its full benefits, as passive NMES alone might not significantly alter postprandial glucose responses [32,72]. Recently, we have observed that 30 min of passive NMES, performed alternating 30 s of work with 60 s of rest, did not elicit any change on the postprandial glucose response in healthy individuals [32]. In contrast, performing voluntary muscle contractions in the lower limbs markedly decreased the glucose peak [32]. Furthermore, the advantageous outcomes of combining resistance exercise with NMES have also been observed in people with T2D. Indeed, 20 min of NMES combined with whole-body resistance exercises led to a notable attenuation of the post-meal glucose response [72]. This suggests that incorporating NMES into a resistance training regimen could offer a beneficial alternative for improving postprandial glucose control, especially for those unable to engage in traditional exercise routines. However, to ensure a practical and comfortable approach, careful consideration of frequency and duration of the stimulation must be given.

Aerobic and resistance exercises exhibit similar efficacy on the post-meal glucose response, offering valuable insights for both healthy individuals and those managing diabetes. For instance, engaging in 30 min of moderate-intensity aerobic exercise has been found to reduce overall glycemic responses as effectively as a matched-duration resistance exercise in healthy subjects [34]. This extends to those with T2D, where 45 min of pre-meal cycling and resistance exercise yielded comparable effects [73]. Furthermore, our research supports these findings, indicating that for people with T2D, 30 min of walking can lower post-meal glucose levels similarly to 15 min of resistance exercise. However, it was observed that aerobic exercise might offer a greater stimulus for mitigating the post-meal glucose peak [24], whereas resistance contributes to more stable glycemic levels over time [24].

The integration of aerobic and resistance exercises into a single regimen has been also investigated. Research has demonstrated that a combination of both exercise types offers superior benefits to the two types alone for improving 24 h glucose control [12,74]. However, when focusing solely on the post-meal glucose response, the benefits of the combination appear similar to aerobic or resistance exercise performed separately [23,24].

The sequence in which aerobic and resistance exercise are performed, when combined, may influence their effectiveness in glucose control in individuals with diabetes. Previous reports have indicated that, in individuals with type 1 diabetes, performing aerobic exercise before the resistance bout resulted in a more pronounced hypoglycemic effect compared to the reverse order [75]. These effects were attributed to the increased secretion of growth hormone during resistance exercise, which moderated the decline in glucose levels during the subsequent bout [76]. Recently, we have reported comparable outcomes in individuals with T2D, indicating a more significant decrease in the post-meal glucose peak following 30 min of combined aerobic–resistance exercise compared to the reverse sequence, highlighting the potential for strategically ordered exercise routines to enhance glycemic control in this population [24].

Hence, exercise prescriptions should consider various factors, including individuals’ time and space availability, cognitive and physical conditions, and personal preferences to promote adherence to the exercise program. While the overall reductions in post-meal glucose responses following aerobic exercise, resistance exercise, or a combination of both exercise types are comparable, individuals with T2D might benefit more from either engaging in aerobic exercise alone or starting with aerobic exercise before moving on to resistance training sessions to achieve a more pronounced mitigation of postprandial glucose excursions. In addition, for situations where traditional exercise may not be feasible, practical, or desired, incorporating simple movements or activities can be adopted to mitigate the glycemic response to a meal, even while seated. It has indeed been demonstrated that even simple “leg fidgeting” performed at regular intervals during the postprandial period can be effective in attenuating the postprandial glycemic and insulinemic response in obese individuals [77]. Furthermore, this strategy may counteract endothelial dysfunction induced by prolonged periods of sitting [78]. Similarly, the repeated contraction of a small amount of muscle mass for several hours post-meal, through so-called “soleus pushups”, appears to lead to a significant reduction in postprandial insulin and glucose concentrations in healthy individuals [79]. These minimal yet effective strategies can offer practical alternatives for improving glucose control, even in sedentary settings, underscoring the flexibility and adaptability required in managing diabetes effectively. Table 2 summarizes the key findings from the studies comparing different exercise types.

## 4. What Should Be the Exercise Duration and Intensity?

Exercise volume plays a crucial role in session planning, necessitating customized adjustments in duration and intensity to suit individual capabilities, yet guidelines on how to prescribe exercise volume for enhancing post-meal glucose control are still vague. The latest physical activity guidelines for individuals with T2D recommend engaging in 45 min of exercise at any intensity for improving post-meal glucose management [20]. However, time constraints pose a significant challenge to maintain high adherence to exercise. Similarly, high-intensity exercise might be challenging for some individuals, particularly when conducted shortly after a meal, which may lead to increased gastrointestinal disturbances, in addition to the increase in hepatic glucose production [81,82]. Hence, more detailed guidelines are required for ensuring a correct exercise volume prescription to provide an effective stimulus for enhancing postprandial glucose responses.

Duration and intensity should be tailored to enable individuals to complete the exercise session. Research covering a broad spectrum of exercise durations has shown that activities ranging from 10 to 120 min can positively affect post-meal glucose responses in both healthy individuals and those with T2D [34,38,83,84,85,86,87]. Notably, sessions lasting between 30 and 60 min has been consistently associated with significant improvements in postprandial blood glucose levels [60,62,70,86,87,88]. Such exercise durations have led to notable enhancements in overall glucose management, including reductions in post-meal glucose peaks and improvements in glycemic variability. This occurred for both light-to-moderate intensity exercises conducted after meals with varied compositions [23,24,31,32,34,38,60,62,88]. Our findings align with this, showing that engaging in 30 min of moderate-intensity aerobic exercise improved postprandial glucose responses similarly to a 45 min session [23]. Additionally, shorter durations of exercise, such as 10–15 min at light intensity, have demonstrated beneficial effects on glucose peaks and blood glucose levels comparable to longer durations of 30–40 min in healthy individuals [89,90].

Similar results were also observed in T2D patients. In fact, it has been shown that engaging in 15 min of light-to-moderate walking after each meal effectively reduces the postprandial response to the meal [41,91]. Likewise, 10 min of exercise yielded significant improvements in the post-meal glucose response, showing comparable effects to those observed with a 30 min session [83]. Additionally, engaging in 20 min of self-paced walking after a meal was equally effective as longer durations of 40 or 60 min in reducing postprandial blood glucose levels in individuals with T2D [92].

Collectively, these studies suggest that even brief exercise sessions at moderate intensities can serve as a viable stimulus for improving the glucose response to meals in both healthy individuals and those with T2D. Therefore, once the minimum dose is reached, the exercise duration has a smaller impact on postprandial glucose responses compared to other crucial parameters, such as timing. Table 3 summarizes the key findings from the studies comparing different exercise durations.

Exercise intensity should be tailored not only in terms of exercise duration, but also to personal capabilities, health conditions, and preferences in order to promote adherence to postprandial exercise sessions. Some people may prefer short bouts of high-intensity exercise for higher enjoyment and as a time-saving strategy [93]. Indeed, previous studies have widely shown that high-intensity interval exercise is effective in improving post-meal glucose responses, daily glycemic variability, and overall 24 h glucose control [80,94]. These effects are comparable to those seen with continuous light- and moderate-intensity exercise in both healthy populations and populations with diabetes [28,95,96]. For example, Shambrook et al. (2018) found no difference in the post-meal glucose response among healthy individuals when they engaged in 30 min of postprandial continuous cycling starting 30 min after breakfast at either light (35 ± 7% VO_2_ reserve), moderate (48 ± 8% VO_2_ reserve), or vigorous intensity intervals (10 × 1 min bouts at 80% VO_2_ reserve with 2 min intervals at 31 ± 12% VO_2_ reserve) [62]. Conversely, Achten et al. (2003) reported that lower intensities (20 min of post-meal cycling at 20% or 40% of peak power output) were more effective in reducing post-meal glucose responses compared to higher intensities (cycling at 80% of peak power output) [97]. Light-to-moderate-intensity exercise after a meal may also offer a lower likelihood of gastrointestinal discomfort. Additionally, high-intensity exercise, due to the adrenergic response, may increase the rate of glucose appearance, resulting in the undesirable effect of blood glucose levels rising [12,42]. However, it has been previously shown that high-intensity interval exercise may increase glucose tolerance and insulin sensitivity, resulting in an enhanced glucose disposal to skeletal muscles, especially when performed after meals [43].

Therefore, the picture emerging from the current knowledge emphasizes the need to tailor exercise prescriptions to individual preferences and requirements. Notably, even a 15 to 30 min continuous exercise session can be effective in improving postprandial glycemia in both healthy individuals and those with T2D. Considering the duration, it is advisable to regulate intensity, favoring a light-to-moderate level when exercise is performed soon after meals. This approach not only supports more effective glucose management, but also encourages long-term exercise adherence by enhancing comfort, particularly due to potential gastrointestinal conditions at higher exercise intensities. Table 4 summarizes the key findings from the studies comparing different exercise intensities.

## 5. Activity Breaks: An Effective Exercise Modality

The rise in sedentary behaviors over recent years has posed significant challenges to maintaining optimal glucose control, especially post-meals. While continuous exercise effectively manages post-meal glycemia, its sustained execution can be challenging or undesirable for many. Dividing exercise into shorter bouts could provide an effective alternative for eliciting a positive impact on cardiometabolic variables and specifically for improving the post-meal glucose response. Studies have indicated that breaking up exercise into shorter segments, such as 15 min intervals around meals, may elicit similar or greater effects on meal-induced glucose responses in individuals with prediabetes or T2D compared to a singular longer session (45 min) performed in the morning or afternoon [98]. Similarly, these results were observed in healthy individuals, indicating improved postprandial glucose control with just 10 min of exercise before a meal [99]. Some evidence suggests that even shorter activity breaks might yield comparable or greater effects on post-meal glucose responses than prolonged exercise [23]. An interesting finding is that a combination of short activity breaks with a continuous exercise routine may be more effective than either modality alone. For example, combining 3 min activity breaks every 30 min of sitting with a 20–30 min continuous exercise routine significantly reduced the glucose responses to breakfast and lunch in obese individuals compared to continuous exercise without breaks [100].

Prescribing activity breaks requires consideration of several parameters, much like with continuous exercise, such as the type, intensity, duration, and frequency of the bouts. Studies have highlighted the effectiveness of various types of activity breaks in improving postprandial glucose responses. Individuals with cardiometabolic disorders, for instance, benefited from performing two sets of 15 repetitions of different resistance exercises every hour over a 4 h period post-meal [101]. Similarly, people with T2D also saw improvements from engaging in 6 min resistance exercises every 60 min of sitting for 7.5 h following their first meal of the day [102]. However, contrasting findings exist regarding the effects of resistance activity breaks in healthy individuals, with some noting significant benefits [103,104] and others observing no change in glucose responses [105,106]. Importantly, activity breaks can also consist of simple bodyweight exercises [104], which offer a versatile option in various everyday life contexts such as, for example, for avoiding prolonged sitting during the evening hours [107]. Activities like walking [23], leg cycling [108], and seated upper body movements have been proven effective for attenuating postprandial glycemic and insulinemic responses, particularly in obese individuals [109]. Stair climbing for short periods has also been shown to be an effective and practical exercise for breaking sedentary time [110,111], making it a viable option for workplace wellness programs [112]. Conversely, standing as an activity break type has yielded mixed results in postprandial glucose control and in any case exerts a lower effect compared to walking [113].

Studies examining the intensity of activity breaks have revealed that both light and moderate intensities can effectively mitigate post-meal glucose responses in individuals with metabolic disorders. For example, two minutes of light- or moderate-intensity walking breaks every 20 min of sitting significantly attenuated postprandial glucose responses in different population groups [114,115]. This effect extends to vigorous-intensity exercise, where two minutes of walking every 20 min of sitting, performed at moderate or vigorous intensities, significantly improved post-meal glucose responses in overweight and obese individuals [116]. Furthermore, there is evidence that vigorous-intensity activity breaks provided a better glycemic control when overweight/obese young men underwent activity breaks at higher compared to lower intensities [117].

Shorter bouts of activity may similarly impact postprandial glycemia compared with longer matched-intensity bouts. As a matter of fact, recent findings suggest brief walking breaks, lasting either 2.5 or 5 min, every 15 min of sitting, can similarly mitigate post-meal glucose excursions and improve glycemic responses over a 3 h period in healthy individuals [23].

While adjusting exercise bout duration and intensity is crucial to ensure tolerability for individuals based on their abilities and preferences, another essential factor to consider is the frequency of breaks, which appears to influence the post-meal glucose response. Several studies have shown a positive correlation between higher frequencies of activity breaks and greater improvements in postprandial glycemia in both healthy and diabetic populations [118,119,120]. While there is some variability in findings, with some studies indicating comparable effects regardless of break frequency, recent evidence suggests that more frequent breaks, such as exercising for 3 min every 15 min of sitting [120], or for 5 min every 30 min [121], might represent the optimal strategy for improving the post-meal glucose response.

In conclusion, activity breaks present a significant opportunity for both preventive and therapeutic intervention in managing postprandial glucose levels, particularly for those at risk of or with T2D. The mechanism underlying their effectiveness likely involves the substantial stimulation exerted by muscle during repeated short bouts of exercise, which may enhance glucose uptake and facilitate a steady reduction in blood sugar levels. Notably, the avoidance of a glucose rebound after the cessation of exercise sessions underscores the benefits of frequent muscle activation throughout the postprandial period [23]. However, further studies are required to elucidate this aspect, evaluating how activity breaks influence the post-meal glucose levels. Interestingly, the potential role of gut hormones in modulating the postprandial response, enhanced by intermittent exercise like walking, introduces an additional layer of complexity [122]. Incorporating the activity breaks-based approach may serve as a complement to traditional continuous exercise routines or as a standalone strategy to provide a flexible, accessible means of glucose control for individuals who may find prolonged exercise sessions challenging, offering similar if not even greater benefits. Table 5 summarizes the key findings from the studies comparing different activity break protocols.

## 6. Conclusions

**Figure 1:**
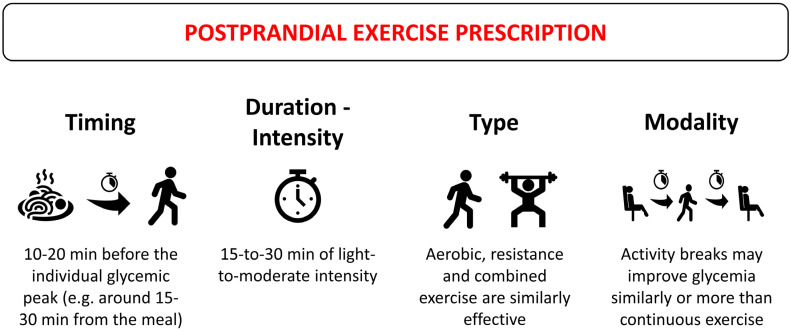
General recommendations for postprandial exercise prescription for post-meal glucose management.

While it is established that physical exercise plays an essential role in promoting postprandial glycemic control, to date, recommendations are quite general, lacking the specificity needed for tailoring exercise prescriptions. Evidence suggests the efficacy of moderate-intensity exercise conducted in the postprandial period for acutely lowering post-meal glucose levels, with added benefits observed when exercise starts soon after the meal (around 10–20 min before the expected glucose peak for healthy individuals, and within 15–30 min from the onset of the meal for people with diabetes). A session duration of 30 min of moderate-intensity exercise may be adequate to enhance postprandial glucose control (Figure 1). High-intensity exercise may also provide benefits, although it is more suitable when performed before a meal.

The reduction in the glucose response to a meal suggests that the type of exercise—whether aerobic, resistance, or a combination of those—should be guided by personal preference, accessibility, and feasibility for the individual. The sequence combining aerobic and resistance exercises could further optimize glycemic control in people with diabetes. Additionally, breaking down the exercise session into shorter activity bouts throughout the postprandial period might be more effective in improving post-meal glucose control in both healthy individuals and individuals with T2D.

Future studies should delve into the mechanisms that underpin the relationship between exercise and postprandial glucose control to better understand how exercise variables—such as timing, type, intensity and duration—specifically influence blood glucose dynamics after a meal. Additionally, exploring further the effects of exercising at different times of the day would further shed light on optimal windows for exercise to maximize glycemic benefits. Further studies are also required for a better understanding of the interplay between exercise and pharmacological treatments for diabetes, in order to elucidate whether a synergistic relationship exists between these interventions, potentially reducing reliance on medication through tailored exercise programs. In addition, further exploration is required to better understand the chronic effects of specific exercise regimens on metabolic health.

In any case, given the diverse ways through which exercise can exert its beneficial effects, an emphasis on personalized exercise prescriptions becomes paramount. A tailored approach that considers an individual’s specific needs, alongside their preferences and desires, ensures that the exercise regimen is not only practical but also enjoyable. This personalization is fundamental for promoting adherence over time and consequently enhancing its therapeutic effectiveness.

## Figures and Tables

**Table 1:** Summary of representative studies comparing different exercise timings.

Reference	Population	Meal	Exercise Protocols *	Post-Meal Glucose Response
**Bellini et al. (2021) [23] ^a^**	27 H in two of six studies (Study 1: 14, 8 M and 6 F, 25 ± 2 years old; Study 4: 13, 8 M and 5 F, 23 ± 1 yrs)	MM: ~75% CHO of total EI (1 g of CHO per kg of BW).	Study 1: Ti: 30 min vs. 60 min vs. 90 min after the meal T: A (walking) D: 60 min I: 120 spm Study 4: Ti: 45 min pre-meal vs. 15 vs. 30 min after the meal T: A (walking) D: 30 min I: 120 spm	Study 1: ↓ with all timings. Study 4: ↓ with 15 and 30 min post-meal, greater effects at 15 min.
**Heden et al. (2015) [27] ^a^**	13 T2D (5 M and 8 F, 48.5 ± 11.9 years old)	Three MMs: ~50% CHO.	Ti: 20–30 min before vs. 45 min after the meal T: R D: 3 × 10 reps I: 10 RM	↓ with pre- and post-meal exercise.
**Hatamoto et al. (2017) [30] ^a^**	11 H (11 M, 23 ± 2 years old)	Three MMs: 113.8 ± 16.6 g (69 ± 3% of EI) CHO for B, 104.5 ± 0.1 g (57 ± 2% of EI) CHO for L and 131.6 ± 11.7 (58 ± 2% of EI) CHO for D.	Ti: 30 min before vs. after each meal T: A (jogging) D: 20 × 1 min with 30 s of rest (60 min in total) I: Individuals’ LT	↓ with post-meal exercise, more than pre-meal.
**Nygaard et al. (2017) [33] ^a^**	12 IGT (8 M and 4 F, 65 ± 8 years old)	MM: 1 g of CHO per kg of BW (74 ± 9 g) for B, 52 ± 19 g CHO for L, 51 ± 17 g CHO for D, and 59 ± 33 g CHO for the evening meal.	Ti: 1.5 h before vs. 30 min after B T: A (walking) D: 60 min I: 8% treadmill inclination, individual speed (12 on Borg’s RPE scale)	↓ with post-meal exercise.
**Solomon et al. (2020) [34] ^a^**	48 H (Group 1: 16, 11 M and 5 F, 31 ± 11 years old; Group 2: 16, 5 M and 11 F, 24 ± 7 years old; Group 3: 9 M and 7 F, 29 ± 12 years old)	MM: 57% CHO of EI (71 g).	Ti: Immediately before vs. after the end of the meal vs. 30 min after the end of the meal T: Standing (Group 1) vs. walking (Group 2) vs. R (Group 3) D: 30 min (Groups 1 and 2) 3 × 10 repetitions (Group 3) I: Self-selected brisk pace (Group 2) and BW (Group 3)	↓ with immediate post-meal exercise.
**Colberg et al. (2009) [35] ^b^**	12 T2D (6 M and 6 F, 61.4 ± 2.7 years old)	MM: 43–54 g CHO.	Ti: Immediately before vs. 15–20 min after the meal T: A (walking) D: 20 min I: Self-selected pace (moderate)	↓ with post-meal exercise.
**Yoko et al. (2021) [36] ^a^**	11 H (5 M and 6 F, 42.7 ± 9.4 years old)	MM: 40 g of CHO.	Ti: 20 min before vs. 40 min after the meal. T: A (walking) vs. R D: 20 min I: 4–6 km/h (A) and BW (R)	↓ with post-meal A.
**Reynolds and Venn (2018) [37] ^a^**	78 H (Group 1: 38, 6 M and 32 F, 21.4 ± 1.35 years old; Group 2: 40, 10 M and 30 F, 22.3 ± 5.16 years old)	MM: 50 g of CHO.	Ti: 15 min (Group 1) vs. 45 min (Group 2) after the meal T: A (cycling) D: 10 min I: 40 rpm, no resistance	↓ with timing set at 45 min after the meal.
**Zhang et al. (2021) [38] ^a^**	20 H (20 M, 23.0 ± 4.26 years old)	MM: 73% CHO of EI (1 g of CHO per kg of BW).	Ti: At the individuals’ glucose peak vs. 20 min before the individuals’ glucose peak T: A (walking) D: 30 min I: 50% VO_2max_	↓ with both timings, greater effects before the peak.
**Huang et al. (2018) [40] ^a^**	26 T2D (12 M and 14 F, 53.8 ± 8.6 years old)	Four MMs: 40–50% CHO of daily EI. B consisted of 30% of TDEI.	Ti: 30 min vs. 60 min vs. 90 min after B T: A (cycling) D: 6 × 1 min + 3 min of recovery (27 min in total) I: 85% W_max_ (active phase) and 40% W_max_ (recovery)	↓ with all timings, greater effects at 30 min.
**Chang et al. (2023) [47] ^a^**	35 T2D (Group 1: 19, 10 M and 9 F, 65.9 ± 6.1 years old; Group 2: 16, 8 M and 8 F, 62.3 ± 7.4 years old)	Normal dietary habits.	Ti: 30 min before (Group 1) vs. 90 min after (Group 2) the individuals’ glucose peak T: A (self-selected) D: 22 min/day for 2 weeks I: Self-selected (moderate)	No changes with both timings.
**Haxhi et al. (2016) [49] ^a^**	9 T2D (9 M, 58.2 ± 6.6 years old)	MM: 55–60% of CHO of EI.	Ti: 40 min after vs. immediately before (1st bout) and 40 min after (2nd bout) the beginning of the meal T: A (walking) D: 40 min vs. 2 × 20 min I: 50% HRR	↓ with split (2 × 20 min bouts) exercise.
**Sacchetti et al. (2021) [50] ^a^**	9 H (9 M, 29 ± 3 years old)	MM: 55–60% of CHO of EI.	Ti: 40 min after vs. 30 min before (1st bout) and 40 min after (2nd bout) the beginning of the meal T: A (cycling) D: 40 min vs. 2 × 20 min I: 65% VO_2max_	↓ with both exercise strategies.

**Notes.** *, protocols are reported as timing (Ti), type (T), duration/volume (D), and intensity (I); ^a^, data are reported as mean ± SD; ^b^, data are reported as mean ± SEM. **Abbreviations.** H, healthy; M, male; F, female; MM, mixed meal; CHO, carbohydrates; EI, energy intake; BW, bodyweight; A, aerobic exercise; spm, steps per minute; ↓, reductions in post-meal glycemia; T2D, type 2 diabetes; R, resistance exercise; RM, repetition maximum; B, breakfast; L, lunch; D, dinner; LT, lactate threshold; IGT, impaired glucose tolerance; RPE, rate of perceived exertion; rpm, rotations per minute; VO_2max_, volume of maximal oxygen uptake; TDEI, total daily energy intake; W_max_, maximal power; HRR, heart rate reserve.

**Table 2:** Summary of representative studies comparing different exercise types.

Reference	Population	Meal	Exercise Protocols *	Main Findings
**Bellini et al. (2021) [23] ^a^**	20 H in two of six studies (Study 2: 10, 5 M and 5 F, 24 ± 3 years old; Study 3: 10, 4 M and 6 F, 24 ± 6 years old)	MM: ~75% CHO of total EI (1 g of CHO per kg of BW).	Study 2: T: A (walking) vs. R vs. AR Ti: 30 min after the meal D: 30 min I: 120 spm and BW or elastic bands Study 3: T: A (walking vs. cycling vs. elliptical exercise) Ti: 30 min after the meal D: 30 min I: 70% HR_max_	↓ with A, R and AR (Study 2) and different A types (Study 3).
**Bellini et al. (2021) [24] ^a^**	8 T2D (3 M and 5 F, 62.6 ± 9.4 years old)	MM: 66% CHO of EI.	T: A vs. AR vs. RA vs. R Ti: 30 min after the meal D: 30 min (A, AR and RA) or 15 min (R) I: 100 spm (A bouts) and BW or elastic bands (R bouts)	↓ with all types, greater effects of A and AR.
**Bellini et al. (2023) [32] ^a^**	23 H (Study 1: 12, 5 M and 7 F, 24 ± 3 years old; Study 2: 11, 9 M and 2 F, 27 ± 4 years old)	MM with 1 g of CHO per kg of BW (Study 1: 69.56 ± 14.97 g, 79.48 ± 4.00% of EI; Study 2: 69.64 ± 9.80 g, 74.51 ± 5.16% of EI).	Study 1: T: Walking vs. stepping vs. isometric wall squat Ti: 15 min after the meal D: 30 min I: 120 spm (walking and stepping) or BW (isometric wall squat) Study 2: T: Walking vs. passive NMES vs NMES + voluntary contraction Ti: 15 min after the meal D: 30 min I: 120 spm (Walking) or 30 Hz (NMES)	Study 1: ↓ with stepping and walking. Study 2: ↓ with walking and NMES with voluntary contraction.
**Solomon et al. (2020) [34] ^a^**	48 H (Group 1: 16, 11 M and 5 F, 31 ± 11 years old; Group 2: 16, 5 M and 11 F, 24 ± 7 years old; Group 3: 9 M and 7 F, 29 ± 12 years old)	MM: 57% CHO of EI (71 g).	T: Standing (Group 1) vs. walking (Group 2) vs. R (Group 3) Ti: Immediately before vs. after the end of the meal vs. 30 min after the end of the meal D: 30 min (Groups 1 and 2) 3 × 10 repetitions (Group 3) I: Self-selected brisk pace (Group 2) and BW (Group 3)	↓ with all exercise types.
**Yoko et al. (2021) [36] ^a^**	11 H (5 M and 6 F, 42.7 ± 9.4 years old)	MM: 40 g of CHO.	T: A (walking) vs. R Ti: 20 min before vs. 40 min after the meal. D: 20 min I: 4–6 km/h (A) and BW (R)	↓ with A.
**Takaishi and Hayashi (2017) [66] ^a^**	7 IGT and 7 T2D (9 M and 5 F, 60.9 ± 11.2 years old)	MM: 106.5 g of CHO.	T: SCD vs. A (cycling) Ti: 90 min after the meal D: 8–10 reps of 21 steps (SCD) and 5–7 min (cycling) I: 60–65% HRR and 12–13 on Borg’s RPE scale	↓ with both, greater effects with SCD.
**Holzer et al. (2021) [72] ^a^**	6 T2D (3 M and 3 F, 55.2 ± 7.5 years old)	M: 61.5 g of CHO for B and 77.5 g of CHO for L.	T: R vs. R + NMES vs. A (cycling) Ti: 50 min after the meal D: 20 min (1 × 10–20 reps for 8 exercises for R) I: BW or elastic bands (R), 80 Hz and 4–5 on a 10-points muscle contraction scale (R + NMES) and 50% W_max_	↓ with all exercise types.
**Nakayama et al. (2022) [80] ^a^**	12 H (12 M, 24.3 ± 2.3 years old)	MM: 71 g (70.5% of EI) CHO.	T: R HIIE vs. A (running) Ti: 30 min after the meal D: 11 min and 30 min I: BW and 50% VO_2max_	↓ with both exercise types.

**Notes.** *, protocols are reported as type (T), timing (Ti), duration/volume (D), and intensity (I); ^a^, data are reported as mean ± SD. **Abbreviations.** H, healthy; M, male; F, female; MM, mixed meal; CHO, carbohydrates; EI, energy intake; BW, bodyweight; A, aerobic exercise; R, resistance exercise; AR, combined aerobic and resistance exercise; spm, steps per minute; HR_max_, heart rate maximum; ↓, reductions in post-meal glycemia; T2D, type 2 diabetes; RA, combined resistance and aerobic exercise; NMES, neuromuscular electrical stimulation; IGT, impaired glucose tolerance; SCD, stair climbing and descending; HRR, heart rate reserve; RPE, rate of perceived exertion; rpm, rotations per minute; B, breakfast; L, lunch; W_max_, maximal power output; HIIE, high intensity interval exercise; VO_2max_, volume of maximal oxygen uptake.

**Table 3:** Summary of representative studies comparing different exercise durations.

Reference	Population	Meal	Exercise Protocols *	Main Findings
**Bellini et al. (2021) [23] ^a^**	12 H in Study 5 (6 M and 6 F, 24 ± 2 years old)	MM: ~75% CHO of total EI (1 g of CHO per kg of BW).	D: 30 min vs. 45 min Ti: 15 min after the meal T: A (walking) I: 120 spm	↓ with all durations.
**Van Dijk et al. (2012) [59] ^b^**	30 T2D (30 M, 60 ± 1 years old)	Three MMs and three snacks: 55% CHO of TDEI.	D: 60 min vs. 30 min (day 1) + 30 min (day 2) Ti: 1.5 h after B T: A (cycling) I: 50% W_max_	↓ on day 1 with 60 min.
**Bartholomae et al. (2019) [63] ^a^**	34 H (20 M and 14 F, 26.8 ± 6.0 years old and 24.8 ± 4.5 years old, respectively)	OGTT (75 g of dextrose).	D: 1 min vs. 3 min vs. 10 min Ti: 18 min, 25 min or 27 min after the OGTT T: SCD I: 90–110 spm	↓ with 10 min SCD in both sexes.
**Moore et al. (2020) [65]**	30 H (12 M and 18 F, 23.7 ± 3.0 years old)	MM: 53% CHO of EI	D: 1 min vs. 3 min vs. 10 min Ti: 27 min 25 min and 18 min after the meal T: SCD I: Self-selected pace	↓ with all durations. Greater effects with 3 min and 10 min bouts.
**Lunde et al. (2012) [89] ^a^**	11 H (of which 5 with IGT) (11 F, 44 ± 9.3 years old)	MM: 50 g of CHO.	D: 20 min vs. 40 min Ti: 20 min after the meal T: A (walking) I: Individuals’ speed (slow pace)	↓ with both durations.
**Nygaard et al. (2009) [90]**	13 H (13 F, >50 years old)	MM: 1 g of CHO per kg of BW.	D: 15 min vs. 40 min Ti: 15 min after the meal T: A (walking) I: 9 on Borg’s RPE scale	↓ with both durations.
**Blankenship et al. (2019) [92] ^a^**	30 T2D (14 M and 16 F, 64 ± 8.2 years old)	Three isocaloric MMs: 55.4 ± 6.0% CHO of EI per meal.	D: 20 min vs. 40 min vs. 60 min Ti: 30–60 min after B T: A (walking) I: Individuals’ speed (brisk walking)	↓ with all durations on post-B glucose response.

**Notes.** *, protocols are reported as duration/volume (D), timing (Ti), type (T), and intensity (I); ^a^, data are reported as mean ± SD; ^b^, data are reported as mean ± SEM. **Abbreviations.** H, healthy; M, male; F, female; MM, mixed meal; CHO, carbohydrates; EI, energy intake; BW, bodyweight; A, aerobic exercise; spm, steps per minute; ↓, reductions in post-meal glycemia; T2D, type 2 diabetes; TDEI, total daily energy intake; B, breakfast; W_max_, maximal power output; OGTT, oral glucose tolerance test; SCD, stair climbing and descending; IGT, impaired glucose tolerance; RPE, rate of perceived exertion.

**Table 4:** Summary of representative studies comparing different exercise intensities.

Reference	Population	Meal	Exercise Protocols *	Main Findings
**Aadland and Høstmark (2008) [60] ^a^**	9 H (6 M and 3 F, 37.3 ± 12.2 years old)	MM: 1 g of CHO per kg of BW.	I: 9 on Borg’s RPE scale (very light intensity) vs. 11 on Borg’s RPE scale (light intensity) Ti: Immediately after the meal T: A (cycling) D: 30 min	↓ with both intensities.
**Shambrook et al. (2018) [62] ^a^**	10 H (10 M, 37.3 ± 7.3 years old)	MM: 51 ± 12% CHO of EI.	I: 35 ± 7% VO_2_R vs. 48 ± 8% VO_2_R vs. 10 × 1 min at 80% VO_2_R with 2 min of active recovery at 31 ± 12% VO_2_R Ti: 30 min after the meal T: A (cycling) D: 30 min in total	↓ with all intensities.
**Moreira et al. (2012) [69] ^a^**	10 H (10 M, 50.8 ± 12.0 years old) and 9 T2D (9 M, 47.2 ± 12.4 years old)	MM: 45 g of CHO.	I: 23% 1 RM vs. 46% 1 RM Ti: 2 h after the meal T: R D: 25 min in total (3 × 30 reps or 3 × 16 reps)	↓ with R in both populations.
**Achten and Jeukendrup (2003) [97] ^b^**	8 H (8 M, 26.4 ± 2.9 years old)	OGTT (75 g of glucose).	I: 40% vs. 65% vs. 80% W_max_ Ti: 45 min after the OGTT T: A (cycling) D: 20 min	↓ with all intensities.

**Notes.** *, protocols are reported as intensity (I), timing (Ti), type (T), and duration (D); ^a^, data are reported as mean ± SD; ^b^, data are reported as mean ± SEM. **Abbreviations.** H, healthy; M, male; F, female; MM, mixed meal; CHO, carbohydrates; BW, bodyweight; RPE, rate of perceived exertion; A, aerobic exercise; ↓, reductions in post-meal glycemia; VO_2_R, volume of oxygen uptake reserve; T2D, type 2 diabetes; 1 RM, one repetition maximum; R, resistance exercise; OGTT, oral glucose tolerance test; W_max_, maximal power output.

**Table 5:** Summary of representative studies comparing different activity breaks.

Reference	Population	Meal	Exercise Protocols *	Main Findings
**Bellini et al. (2021) [23] ^a^**	14 H in Study 6 (7 M and 7 F, 23 ± 2 years old)	MM: ~75% CHO of total EI (1 g of CHO per kg of BW).	P: 2 × 15 min vs. 6 × 5 min vs. 12 × 2.5 min every 15 min of sitting T: A (walking) I: 120 spm	↓ with 5 and 2.5 min bouts.
**Francois et al. (2014) [29] ^a^**	9 IGT and T2D (7 M and 2 F, 48 ± 6 years old)	Three MMs: 78 g CHO for B, 71 g CHO for L and 100 g CHO for D.	P: 6 × 1 min with 1 min of recovery 30 min before each meal vs. 30 min continuous performed 30 min before D T: A (walking) I: 90% of HR_max_ and 60% HR_max_	↓ with interval walking
**Hatamoto et al. (2017) [30] ^a^**	11 H (11 M, 23 ± 2 years old)	Three MMs: 113.8 ± 16.6 g (69 ± 3% of EI) CHO for B, 104.5 ± 0.1 g (57 ± 2% of EI) CHO for L and 131.6 ± 11.7 (58 ± 2% of EI) CHO for D.	P:20 × 1 min with 30 s of rest 30 min before or after each meal vs. 3 × 1 min performed every 30 min (20 times in total) T: A (jogging) I: Individuals’ LT	↓ with brief periodic exercise bouts.
**Pettit-Mee et al. (2021) [77] ^b^**	20 Ob (5 M and 15 F, 42 ± 3 years old)	OGTT (75 g of glucose)	P: 2.5 min alternated with 2.5 min of resting for 3 h T: Leg fidgeting I: Individual cadence	↓ with leg fidgeting.
**Reynolds et al. (2016) [83] ^a^**	41 T2D (26 M and 15 F, 60 ± 9.9 years old)	Habitual diet.	P: 30 min continuous vs. 3 × 10 min within 5 min after each meal T: A (walking) I: Not specified	↓ with 10 min bouts before each meal.
**Blankenship et al. (2019) [92] ^a^**	30 T2D (14 M and 16 F, 64 ± 8.2 years old)	Three isocaloric MMs: 55.4 ± 6.0% CHO of EI per meal.	P: 20 min vs. 40 min vs. 60 min or 4 × 1.6 min, 3.3 min or 5 min every 30 min after each meal of the day (12 bouts in total). T: A (walking) I: Individuals’ speed (brisk walking)	↓ with both continuous and activity breaks after B.
**Shambrook et al. (2020) [99] ^a^**	10 H (8 M and 2 F, 50 ± 12.6 years old)	Three MMs: 55.2 ± 18.5% CHO of EI.	P: 10 min after each meal vs. 30 min after D T: A (walking) I: 55–70% HRR	No differences in post-D glucose control with both sessions.
**Wheeler et al. (2020) [100] ^a^**	67 Ov and Ob (32 M and 35 F, 67 ± 7 years old)	Two MMs: 109.1 ± 17.3 g of CHO for B and 97.4 ± 19.7 g of CHO for L.	P: 30 min after B vs. 30 min after B + 3 min of every 30 min of sitting for 6.5 h T: A (walking) I: 65–75% HR_max_, 3.2 km/h of speed	↑ with both conditions.
**Kowalsky et al. (2019) [101] ^a^**	14 at risk of cardiometabolic diseases (2 M and 12 F, 53.4 ± 9.5 years old)	MM: 55% CHO of EI.	P: 2 × 15 reps every hour of sitting for 4 h T: R I: BW or elastic bands	↓ with activity breaks.
**Homer et al. (2021) [102] ^a^**	23 T2D (13 M and 10 F, 62 ± 8 years old)	Three MMs: 55% CHO of total EI.	P: 3 min every 30 min of sitting vs. 6 min every 60 min of sitting for 8 h T: R I: BW	↓ post-B and -L with 6 min bouts. ↓ post-L with 3 min bouts.
**Climie et al. (2018) [103] ^a^**	9 Ov and Ob (5 M and 4 F, 32 ± 3 years old)	MM: 53–55% CHO of daily EI.	P: 3 min every 20 min of sitting for 3.5 h T: R I: BW	↓ with activity breaks.
**Dempsey et al. (2016) [104] ^a^**	24 T2D (14 M and 10 F, 62 ± 6 years old)	Three MMs: 55–58% CHO of EI.	P: 3 min every 30 min of sitting for 7 h T: A (walking) vs. R I: 3.2 km/h and BW	↓ with both A and R.
**Gillen et al. (2021) [105] ^a^**	14 H (7 M and 5 F, 24 ± 5 years old)	Two MMs: 56 ± 12 g (55% of EI) of CHO for B and 84 ± 18 g (55% of EI) of CHO for L.	P: 2 min for A vs. 1 min for chair stands every 30 min of sitting for 7.5 h T: A (walking) vs. chair stands with calf raise I: 3.1 mph and BW	No effects on post-meal glycemia with both protocols.
**Charlett et al. (2021) [106] ^a^**	12 H (5 M and 7 F, 25 ± 6 years old)	Two MMs: 57% CHO of EI for B and 51% CHO of EI for L.	P: 3 min every 30 min of sitting for 5 h T: R I: BW	↑ with activity breaks.
**Gale et al. (2023) [107] ^a^**	10 H-NW (4 M and 6 F, 23.5 ± 4.2 years old), 10 Ov (1 M and 9 F, 25.8 ± 5.8 years old) and 10 Ob (3 M and 7 F, 26.8 ± 5.8 years old)	Two MMs: 114.8 21.3 g (59% of EI) of CHO.	P: 3 min every 30 min of sitting for 4 h T: R I: BW	↓ with activity breaks in all groups. Greatest reductions in H-NW.
**Engeroff et al. (2022) [108] ^a^**	18 H (18 F, 25.6 ± 2.6 years old)	Free-portion size MM: 51% CHO of EI.	P: 30 min continuous vs. sitting interrupted by 5 breaks of 6 min for 4 h T: A (cycling) I: 70% VO_2max_	No effect on post-meal glycemia.
**McCarthy et al. (2017) [109] ^a^**	13 Ob (6 M and 7 F, 66 ± 6 years old)	Two MMs: 51% CHO of EI.	P: 5 min every 30 min of sitting for 7.5 h T: A (arm ergometer) I: 15-35 W	↓ with arm exercise.
**Rafiei et al. (2021) [110] ^a^**	12 H (Study 1: 12 M, 22.8 ± 4.3 years old) and 11 Ov and Ob (Study 2: 3 M and 8 F, 50.2 ± 14.3 years old)	Three MMs: 97 g of CHO.	P: In both studies: 8 × 15–30 s every hour for 9 h T: SCD I: Quickest pace possible	No effects on post-meal glycemia in both H and Ov and Ob.
**Cho et al. (2020) [111] ^a^**	12 H (7 M and 5 F, 23.5 ± 2.9 years old)	MM: 83 g of CHO.	P: 5 min every hour for 4 h T: SCD I: 66% HRR and 15 on Borg’s RPE scale	↑ with SCD.
**Bailey et al. (2016) [114] ^a^**	13 H (6 M and 7 F, 26.6 ± 8.5 years old)	Mixed drink: 75 g of CHO.	P: 2 min every 20 min of sitting for 5 T: A (walking) I: 3.2 km/h vs. 5.8–6.4 km/h	↓ with both protocols. Greater effects with moderate-intensity.
**Dunstan et al. (2012) [115] ^a^**	19 Ov and Ob (11 M and 8 F, 53.8 ± 4.9 years old)	Mixed drink: 75 g of CHO.	P: 14 × 2 min every 20 min of sitting for 5 h T: A (walking) I: 3.2 km/h vs. 5.8–7.9 km/h	↓ with both intensities.
**Bhammar et al. (2017) [116] ^a^**	10 Ov and Ob (5 M and 5 F, 32 ± 5 years old)	Five MMs: 130 g (77% of EI) of CHO for B, 68 g (53% of EI) of CHO for L, 148 g (58% of EI) of CHO for D and 19 g of CHO for S.	P: 30 min continuous vs. 21 × 2 min every 20 min of sitting vs. 8 × 2 min every hour for 9 h T: A (walking) I: 65–75% HR_max_ (230 kcal of EE) vs. 3.0 mph (240 kcal of EE) vs. 79 ± 4% HR_max_ (140 kcal of EE)	↓ with all protocols.
**Hatamoto et al. (2021) [117] ^a^**	9 Ov and Ob (9 M, 21.1 ± 0.9 years old)	Two MMs: 99 g (55% of EI) of CHO after B and 141 ± 17 g (55 ± 2% of EI) of CHO after L.	P: 2 min vs. 200 s vs. 75 s every 30 min of sitting for 8 h T: A (running) I: At LT vs. 60% LT vs. OBLA	↓ with exercise at LT and OBLA intensities.
**Maylor et al. (2019) [118] ^a^**	14 H (14 F, 33.8 ± 13.4 years old)	Two MMs: 43.1 ± 8.1 g (58% of EI) of CHO for the 1st, 57.6 ± 10.0 g (46% of EI) of CHO for the second. In addition, an ad libitum meal was consumed.	P: 2 min every 30 min of sitting vs. 10 min every 170 min of sitting for 7.5 h T: A (walking) I: 65% VO_2peak_	No effects for both conditions on post-meal glycemia.
**Paing et al. (2019) [120] ^a^**	12 T2D (8 M and 4 F, 60 ± 11 years old)	Four MMs: 50–53.7 g of CHO for B, 75 g of CHO for L, 50.1–55.6 g of CHO for D and 10–13.1 g of CHO for S.	P: 3 min every 60 min, 30 min or 15 min of sitting for 7 h T: A (walking) I: 3.2 km/h	↓ with higher activity breaks frequency (i.e., every 15 min).
**Duran et al. (2023) [121] ^a^**	9 H (5 M and 4 F, 57 ± 8.6 years old)	Two MMs: 55–58% CHO of EI.	P: 1 min vs. 5 min every 30 vs. 60 min of sitting for 8 h T: A (walking) I: 2 mph and 0% of treadmill slope	↓ only with 5 min breaks every 30 min.

**Notes.** *, protocols are reported as protocol (P), type (T), and intensity (I); ^a^, data are reported as mean ± SD; ^b^, data are reported as mean ± SEM. **Abbreviations.** H, healthy; M, male; F, female; MM, mixed meal; CHO, carbohydrates; EI, energy intake; BW, bodyweight; A, aerobic exercise; spm, steps per minute; ↓, reductions in post-meal glycemia; IGT, impaired glucose tolerance; T2D, type 2 diabetes; B, breakfast; L, lunch; D, dinner; HR_max_, heart rate maximum; LT, lactate threshold; Ob, obese; OGTT, oral glucose tolerance test; HRR, heart rate reserve; Ov, overweight; ↑, increases in post-meal glycemia; R, resistance exercise; mph, miles per hour; H-NW, healthy normo-weight; VO_2max_, volume of maximal oxygen uptake; SCD, stair climbing and descending; RPE, rate of perceived exertion; S, snack; EE, energy expenditure, OBLA, onset of blood lactate accumulation; VO_2peak_, volume of peak oxygen uptake.
